# On Intensive Late Holocene Iron Mining and Production in the Northern Congo Basin and the Environmental Consequences Associated with Metallurgy in Central Africa

**DOI:** 10.1371/journal.pone.0132632

**Published:** 2015-07-10

**Authors:** Karen D. Lupo, Dave N. Schmitt, Christopher A. Kiahtipes, Jean-Paul Ndanga, D. Craig Young, Bernard Simiti

**Affiliations:** 1 Department of Anthropology, Southern Methodist University, Dallas, Texas, United States of America; 2 Centre Universitaire de Recherche et de Documentation en Histoire et Archeologie Centraficaines, Université de Bangui, Bangui, Central African Republic; 3 Far Western Anthropological Research Group, Carson City, Nevada, United States of America; University of California Davis, UNITED STATES

## Abstract

An ongoing question in paleoenvironmental reconstructions of the central African rainforest concerns the role that prehistoric metallurgy played in shaping forest vegetation. Here we report evidence of intensive iron-ore mining and smelting in forested regions of the northern Congo Basin dating to the late Holocene. Volumetric estimates on extracted iron-ore and associated slag mounds from prehistoric sites in the southern Central African Republic suggest large-scale iron production on par with other archaeological and historically-known iron fabrication areas. These data document the first evidence of intensive iron mining and production spanning approximately 90 years prior to colonial occupation (circa AD 1889) and during an interval of time that is poorly represented in the archaeological record. Additional site areas pre-dating these remains by 3-4 centuries reflect an earlier period of iron production on a smaller scale. Microbotanical evidence from a sediment core collected from an adjacent riparian trap shows a reduction in shade-demanding trees in concert with an increase in light-demanding species spanning the time interval associated with iron intensification. This shift occurs during the same time interval when many portions of the Central African witnessed forest transgressions associated with a return to moister and more humid conditions beginning 500-100 years ago. Although data presented here do not demonstrate that iron smelting activities caused widespread vegetation change in Central Africa, we argue that intense mining and smelting can have localized and potentially regional impacts on vegetation communities. These data further demonstrate the high value of pairing archeological and paleoenvironmental analyses to reconstruct regional-scale forest histories.

## Introduction

An ongoing and key question in paleoenvironmental research concerns the relative contribution that prehistoric anthropogenic processes played in the formation of contemporary vegetation communities. Paleoenvironmental research conducted over the last three decades in central Africa clearly shows that the Holocene epoch ushered in a period of highly volatile climatic fluctuations that were geographically widespread and influenced forest vegetation distribution and composition [1–7]. Yet questions about the timing, scale and scope of prehistoric anthropogenic impacts on the Central African rainforest have persisted for more than a century [8–17]. Surveys identifying unique botanical features and biotic communities, including the presence of monodominant forests, the prevalence of light-demanding and long-lived trees, low species diversity, forest-savanna mosaics, and endemic species led many to infer a long history of anthropogenic disturbance in the Central African rainforest [16, 17, 18–23]. The current consensus is that rainforests are cultural landscapes where a suite of anthropogenic activities may have depleted, disturbed, and/or maintained some resources and sometimes these activities accelerated changes in forest vegetation resulting from climate change [13, 24, 25].

Metallurgy, along with shifting agriculture, is often nominated as one of the key cultural processes that depleted forests, disturbed local vegetation and potentially initiated long-term ecological consequences [8, 26, 27]. The introduction and spread of metallurgy in prehistoric Africa is a pivotal cultural process implicated in the emergence and maintenance of sociopolitical inequalities, a specialized craft and labor force, the support of complex regional trade systems, and as a catalyst for changes in political, belief, and economic systems [28–33]. The production of iron in forested areas of west and central Africa dates back to 2800–2700 yr BP [34–42]; but see [43–47]. Archaeological sites with evidence of metallurgy in the form of iron implements, furnaces, forges, and/or slag become widespread throughout forested areas of west Africa between 1700–1900 BP [41, 48, 49] and in central Africa between 2,200 and 2,100 BP [50] (cf. [51, 52]). In the historic and ethnographic record, raw metal and formed objects were used as money for commercial transactions, were received as taxes and obligatory payments including bride price, served as symbols of power and wealth, figured prominently into rituals and mythology, and had utilitarian functions as tools and weaponry [53–55]. Despite the introduction of imported iron and metal objects during the historic period, the use and production of indigenously produced raw iron and objects continued and in some areas reached peak production levels [56] well into the 19^th^ century and persisted into the ethnographic record [26, 57]. However, despite the clear cultural, sociopolitical, and spiritual importance of iron, its role in shaping vegetation communities in the Central Africa forest is generally presumed to be negligible [13, 58].

In this paper we report the results of surveys and tests excavations near the village of Bagbaya on the northern edge of the Congo Basin. Our research discovered several large open-pit iron mines and 19 smelting features with most dating to a roughly 100-year time span just prior to colonial occupation. Several additional iron-working features pre-dating these suggest an earlier period of iron production at a smaller scale. The scale and intensity of iron production reflected by these features are unprecedented in this part of Central Africa and show the emergence and existence of a pre-colonial regional-scale trade system that persisted until the ethnohistoric period. Although limited, these data offer a glimpse of the resiliency of these economies even as imported items from the Atlantic coast were penetrating inland. Because the intensity and scale of iron exploitation reflected at Bagbaya manifest an ideal circumstance to investigate how metallurgy influenced local vegetation, we examine the types and frequencies of pollen grains and charcoal microfossils preserved in a local sediment core that spans the time interval before and after intensified production.

### Project Setting

**Fig 1:**
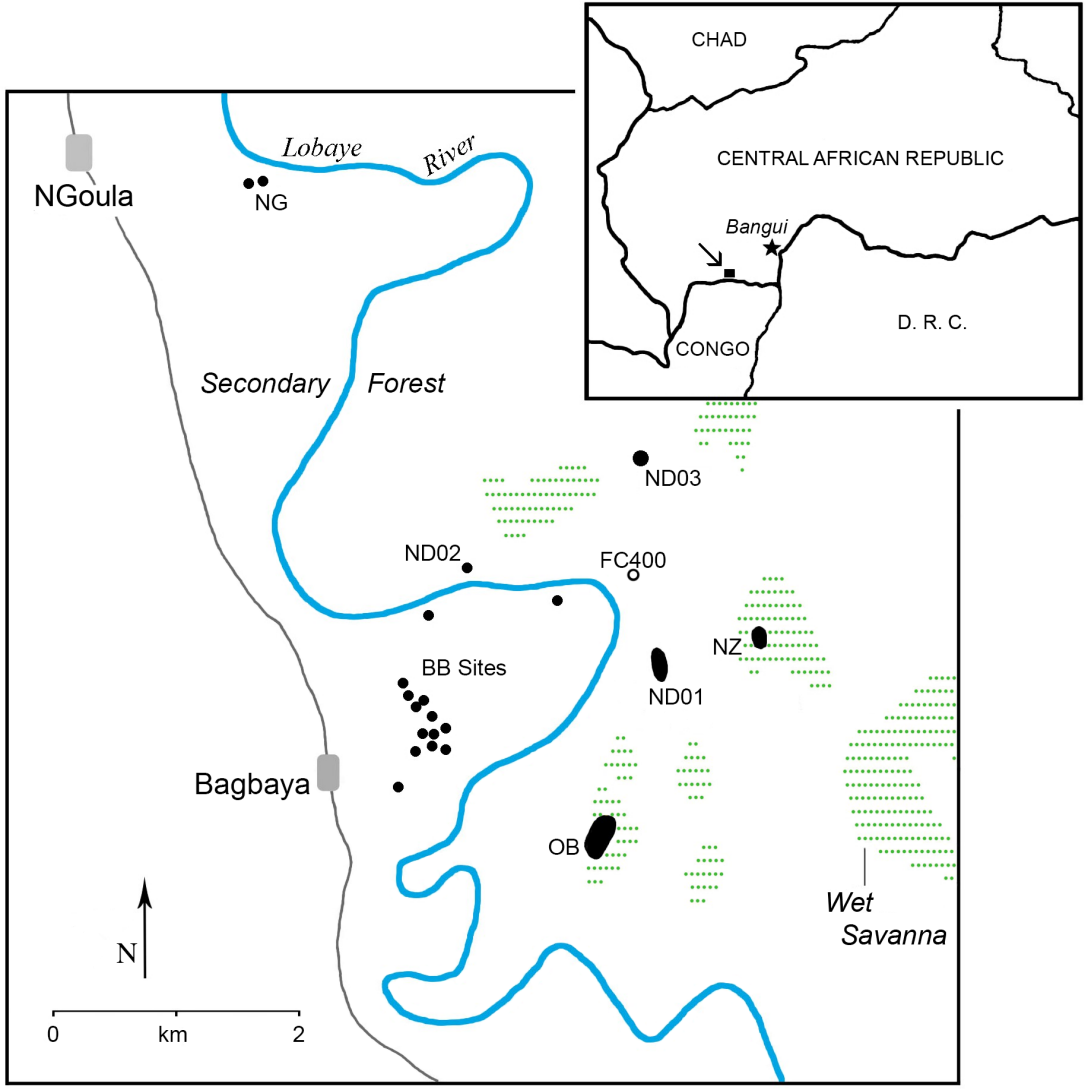
Location of the project area in the eastern NGotto Forest Reserve, southern Central African Republic. Archaeological site locations (labeled) are marked with black dots/ovals and the location of the sediment core (FC400) is marked with an open circle.

The data reported here are from archaeological and paleoecological surveys and excavations conducted on the eastern periphery of the NGotto Forest Reserve (NFR) in the southwestern Central African Republic. Field investigations centered on areas near the village of Bagbaya (which translates to “iron market”) associated with a prominent iron-ore mine known locally as the “Ndanga Grotte” (site ND01; Fig 1). The NFR is a triangular-shaped area that encompasses 3250 m^2^ along the extreme northern edge of the Congo Basin. The area is bounded on the east by the south-flowing Lobaye River and on the west by the Mbaeré River. The Bodingué River marks the approximate southern boundary of the forest and flows east into the Lobaye River. The Lobaye River flows east and southeast and ultimately drains into the Ubangui River which is part of the Congo River system. These major interconnected river systems served as a conduit for economic, demographic and ideological exchange during the pre-colonial and colonial periods.

Although this area is largely classified as a dry, Guinea-Congolian rainforest [17, 59], a mosaic of tropical microenvironments occur, including semi-deciduous rainforests, ephemeral wetlands, riparian swamps with evergreen forests, and wet savannas [60–62]. Large tracts of secondary forest broken by patches of wet savanna are common here and include economically valuable tree species such as Sapele/Sipo (*Entandrophragma cylindricum* and *E*. *utile*), African corkwood (*Musanga cecropioides*), Limba/Korina (*Terminalia superba*), umbrella tree (*Alstonia congensis*), and *Trema orientalis*, commonly referred to as the charcoal-tree.

The area is currently occupied by ethnically distinct Bolemba farming and foraging (i.e., pygmy) populations who speak Mbati, one of the Bantu languages spoken in the Ubangui River region. While the ancestors of some of these people are believed to have entered portions of the Congo Basin some 2500 years ago, oral histories recalled by local residents record the migration of their ancestors from the south (e.g., Democratic Republic of Congo) into this area during the mid-to-late 18^th^ century in response to tribal warfare (cf. [63–65]). According to local tribal historians, their ancestors fled north, crossing the Ubangui River near present day Bangui where their clans dispersed (Nguéta Nixy Honoré, personal communication 2011 (cf. [66]). Some groups went west and southwest and settled in Boda where they experienced intimidation and persecution from local populations. These populations eventually settled further south near the Lobaye River and discovered the ore exposures described here.

Several oral histories collected from villages around the NFR describe the area as having been previously abandoned, except for sparse forager populations. The first village (called “Ekuba” or “Old Bagbaya”) was established in a wet savanna on the east side of the Lobaye River and subsequently moved to a forested area on the river’s west side. At least 17 different clans formed the founding population and were involved in smelting ore. With colonization, the local labor was forcibly diverted to work on rubber and palm oil plantations to the north and east of the NFR and the iron production diminished. After the Kongo-Wara uprising (AD 1928–1931) some of the original occupants of Bagbaya fled into the forest and resettled elsewhere, but many returned. The last known smelter in Bagbaya was a man named Lomé who died in 2007–2008, and one elderly resident recalled seeing iron-smelting in 1948–1950.

## Materials and Methods

Fieldwork was undertaken in 2011. Sites were identified by pedestrian survey, through interviews with local villagers, and by reference to a large, hand-drawn heritage map created by representatives from all of the villages in and adjacent the NFR. Given the thick ground cover of leaves and other forest debris, surface survey was largely restricted to selected forest paths, cleared village areas and agricultural fields, wet savannas, and by judgmental inspection of exposed profiles in stream beds, diamond mines, and brick and trash pits. Sites were mapped using a hand-held GPS unit and selected sites were tested using 1x1-m and 1x2-m units that were hand excavated with picks, shovels, and trowels. Excavated deposits were hand sorted and examined for artifacts, and samples of sediments were screened through 1/4 in mesh. All charcoal samples were collected *in-situ* from subsurface deposits, notably pieces exposed in stratigraphic profiles, and selected samples were assayed at the Accelerator Mass Spectrometry Laboratory, University of Arizona. Representative artifacts and ecofacts were collected from most sites for analysis. Although many specimens are housed at the Department of Anthropology at Southern Methodist University, other specimens reposited at the Centre Universitaire de Recherche et de Documentation en Histoire et Archeologie in Bangui were stolen or destroyed when the facility was sacked during the coup d’état of 2013 (S1 Table).

Additional data were provided by interviews with local farmers and forest foragers who offered information in the form of oral histories (including the names of iron smelting clans), as well as the location of sacred landmarks, traditional forest paths, and landscape features such as wet savannas. The hand-drawn heritage map provided extensive information on regional sacred properties and hunting areas and, more importantly, it plotted the location of the Ndanga Grotte which served as an impetus for our investigations in the Bagbaya vicinity.

Fieldwork, collections, and analyses were performed under permits issued by Le Secrétaire Général, Université de Bangui (Ordre de Mission No. 008/UB/SG) and Le Directeur, Centre Universitaire de Recherche et de Documentation en Histoire et Archeologie Centraficaines, Université de Bangui (No. 009/UB/R/CURDHACA/.11). All necessary permits were obtained for the described study, which complied with all relevant regulations. Surveys and excavations of heritage properties and interviews with Bagbaya villagers were conducted by verbal consent. Consent scripts and interview protocols for this study were approved by the Institutional Review Board on Human Subjects Research, Washington State University, Pullman, Washington before the study began. We proposed to use verbal rather than written consent because some people in rural Central African contexts cannot read or write (which was the case in Bagbaya) and we did not want to cause any embarrassment. As such, our project domains were presented to a council of elders led by the village chef who conveyed our methods and goals to the village. All aspects of our investigations were verbally approved by each village participant and the names of consenting individuals who provided ancestral or demographic information were recorded in a field notebook.

A sediment core was extracted from a local wetland using a portable Russian peat corer. The organic-rich alluvium was sampled in the field with 1-cm sub-samples taken at 5-cm intervals (n = 40) from the length of the core. Palynological samples were analyzed at Southern Methodist University and were prepared according to standard methods [67] with the addition of a heavy density separation using ethanol and a solution of zinc bromide and hydrochloric acid. Samples were then mounted on slides using glycerine and both pollen and microscopic charcoal were identified using a binocular microscope under 40x magnification. Identifications were made by comparison to images in the African Pollen Database (http://medias.obs-mid.fr/apd/) and published pollen atlases for tropical Africa [68, 69]. The palynological interpretations are based on the analysis and identification of 400 pollen grains in each sample, while microscopic charcoal concentrations were determined by counting charcoal fragments (> 10 microns) encountered over a unit of 20 tracer spores (cf. [70]). Identification of grass cuticles was completed by comparison with published scanning electron micrograph images (e.g. [71]) and by referencing a comparative collection housed in the Department of Earth Sciences, Southern Methodist University. All unprocessed sediment samples and sub-samples are housed at the Department of Anthropology at Southern Methodist University (S1 Table).

## Results

Archaeological reconnaissance in the eastern NFR resulted in the identification of 21 sites (Table 1, Fig 1) including two large, open pit iron-ore quarries, one pile of abandoned iron-ore cobbles adjacent the Lobaye River, and a number of discrete, cone-shaped slag piles that represent detritus mounds associated with smelting. Surveys in two wet savannas revealed two large sites containing scattered slag, furnace remnants, and pottery fragments on and around neighboring low mound features that may represent habitation areas coupled with iron production.

**Table 1:** Selected attributes of archaeological sites discovered in the Bagbaya vicinity.

Site	Type	Location	Contents^a^	Test excavation
BB01	Slag mound	Secondary forest	CE, CH, FP, PN, SG	+
BB02	Slag mound	Agricultural field	FP, SG	-
BB03	Slag mound	Agricultural field	CE, CH, FP, SG	+
BB04	Slag mound	Secondary forest	SG	-
BB05	Slag mound	Secondary forest	CE, CH, FP, PN, SG	+
BB06	Slag mound	Secondary forest	SG	-
BB07	Slag mound	Agricultural field	FP, SG	-
BB08	Ore cluster	Secondary forest	IR	-
BB09	Slag mound	Agricultural field	FP, SG	-
BB10	Slag mound	Secondary forest	FP, SG	-
BB11	Slag mound	Secondary forest	FP, SG	-
BB12	Slag mound	Secondary forest	FP, PN, SG	-
BB13/14	Slag mound	Secondary forest	FP, SG	-
BB15	Slag mound	Secondary forest	SG	-
ND01^b^	Ore exposure/quarry	Secondary forest	CE, CH, FP, IR	+
ND02	Slag mound	Secondary forest	CH, FP, SG	+
ND03	Ore exposure/quarry	Secondary forest	IR	-
NG01	Slag mound	Sec. forest-agricult. field	CE, CH, FP, SG	+
NG02	Slag mound	Sec. forest-agricult. field	FP, SG	-
NZ complex^c^	Low mounds	Wet savanna	CE, CH, FP, QZ, SG	+
OB complex^d^	Slag mounds and low mounds	Secondary forest and wet savanna	CE, CH, FP, SG	+

^a^CE = ceramic pottery sherds; CH = charcoal; FP = furnace pipe (tuyère) fragments; IR = iron ore; PN = palm nuts; QZ = quartz cobbles/spall; SG = slag.

^b^Test excavations were undertaken at the entrance (dripline) of the Feature E adit and along the western edge of Feature G.

^c^Five low mound features were observed; Features NZ03 and NZ05 were subject to test excavations.

^d^Includes two slag mounds (OB01-02) in secondary forest and 10 low mound features (OB03-12) in the neighboring wet savanna. Features OB01, 02, 05, and 06 were subject to test excavations.

### Iron Mines

Quarry sites ND01 and ND03 consist of exposures of iron-rich volcanic deposits extensively modified by prehistoric and historic mining activities. Two additional purported quarry areas were identified by local villagers but could not be further explored. One of these consisted of an isolated ore exposure that was nearly completely buried by sediments, and the second was a monolith that was submerged along the bank of the Lobaye River several kilometers south of ND01. Site ND01 (the Ndanga Grotte) is considered to be sacred by the inhabitants of Bagbaya because Ndanga is the name of one of the first miners who discovered the iron-rich exposures and began to quarry and smelt ore. Scattered mafic basalt boulders and several low outcrops and partially exposed monoliths occur between ND01 and ND03 and suggest that the mines are in the same north-to-south-trending volcanic flow. Both loci represent fissure and collapse structures containing excavated adits and deep bulbous caves, and local portions of the flow contained lenses quartz alluvial pebbles and gravels. A number of the cavern walls and exterior rock faces exhibit clusters of parallel linear grooves that doubtless represent tool marks produced in the extraction of ore.

**Fig 2:**
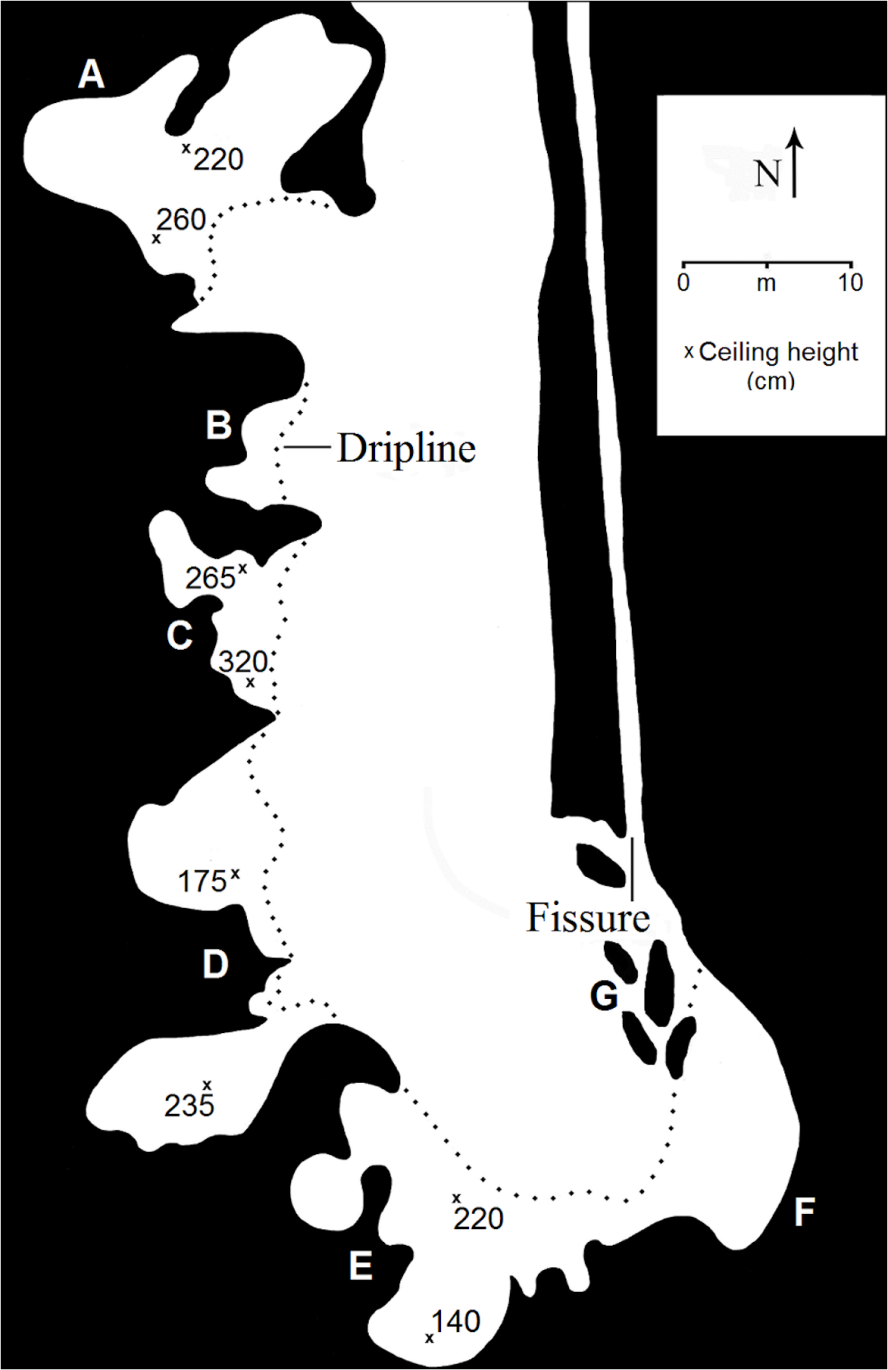
General plan of the southern Ndanga Mines (ND01), Features A-G.

**Fig 3:**
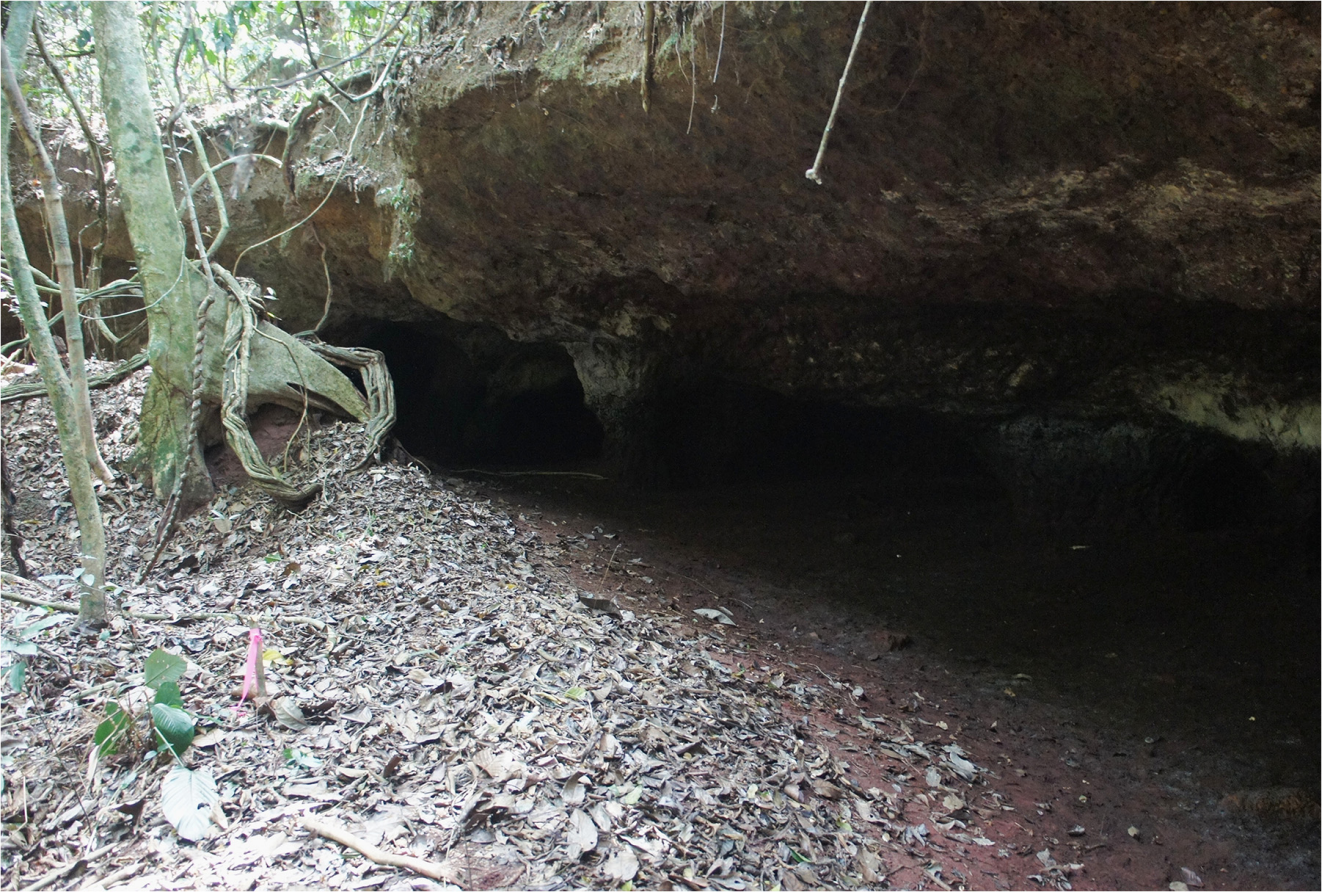
View south/southeast of the Feature E cavern at ND01; photo by KD Lupo.

The southernmost mine, ND01, was recorded in detail and consists of a series of excavated adits and broad caverns (Features A-F; Figs 2 and 3) in the south and west face of a linear crevice that extends to 4–6 m below the surrounding forest floor. Given its proximity to the Lobaye River, the site is often flooded during wet season monsoons and it is a popular fishing location for local villagers. Sediment in the site is partly the result of these seasonal floods and includes fluvial mud, fine-to-medium sand, and lenses of oxidized volcanic gravel from weathering of local exposures and the surrounding regolith. Combined volume estimates for the southern Ndanga mine caverns (Fig 2) suggest that some 800 m^3^ of ore were removed, and an additional ~180 m^3^ of ore deposits appear to have been extracted from four smaller features that mark the northernmost end of the site. These volumes are based solely on the dimensions of the caverns and adits and represent a minimum estimate of the amount of ore removed. Based on solid ore sample weights that equate to approximately 2,900 kg per m^3^, at least 2,840,000 kg of ore deposits have been removed from the site. Limited test excavations inside the dripline of Feature E recovered charcoal directly associated with a few pot sherds and a fragment of ceramic pipe. Radiocarbon assay of the charcoal returned a date of 160 ± 35 yr BP (calibrated age midpoint [cal mid]; AD 1797; Table 2).

**Table 2:** Radiocarbon dates and calibrated (2-sigma) calendrical age estimates on charcoal from sites in the Bagbaya vicinity.

Site	FS no.^a^	Lab no.^b^	Depth (cm) below surface	^14^C age	Calibrated age BP	Calibrated age AD^c^
BB01	30	AA94530	90	168 ± 35	291–0	1804
BB01	31	AA94531	112	207 ± 35	309–0	1795
BB01	29	AA94529	50	215 ± 34	310–0	1795
BB03	36	AA94532	24	148 ± 34	283–0	1808
BB05	41	AA94534	61	187 ± 34	302–0	1799
BB05	32	AA94533	24	231 ± 34	317–144	1719
ND01^d^	3	AA94537	55	160 ± 35	286–0	1797
ND02	76	AA94538	74	242 ± 34	428–145	1664
NG01	74	AA94539	60	217 ± 48	324–0	1788
NZ03	17	AA94541	27	494 ± 34	553–498	1424
NZ03	12	AA94542	33	593 ± 34	653–538	1354
NZ03	23	AA94540	29	706 ± 35	698–562	1320
OB01	67	AA94543	33	152 ± 35	284–0	1808
OB02	49	AA94544	25	210 ± 34	308–0	1796
OB05	54	AA94546	25	131 ± 34	279–8	1806
OB05	53	AA94545	22	188 ± 39	304–0	1798
OB06	63	AA94547	28	131 ± 34	279–8	1806

^a^Field specimen number.

^b^All samples were assayed at the Accelerator Mass Spectrometry Laboratory, University of Arizona, Tucson.

^c^Age midpoint.

^d^Southern Ndanga Mines, Feature E.

Site ND03 (locally known as “Nzingui”) is the northernmost mining locality and was only cursorily visited and documented. According to villagers, ND03 represents the earliest area that was mined by their direct ancestors. It consists of an undulating ~30 x 75-mexposure of iron-ore centered on a large monolith that stands approximately nine meters above the modern ground surface. Extensive mining of this feature has resulted in a large bulbous cavern with a ~north-south tunnel containing a series of excavated pits and adits along its edges. Adits and quarry pits were observed across other portions of ND03 and most exposed surface areas contained abundant mafic gravels and sub-angular cobbles that were commonly oxidized. Although a detailed map was not constructed, the number and extent of quarry pits and caverns/adits suggest that hundreds of cubic meters of iron-ore have been removed from the site.

### Slag Mounds

**Fig 4:**
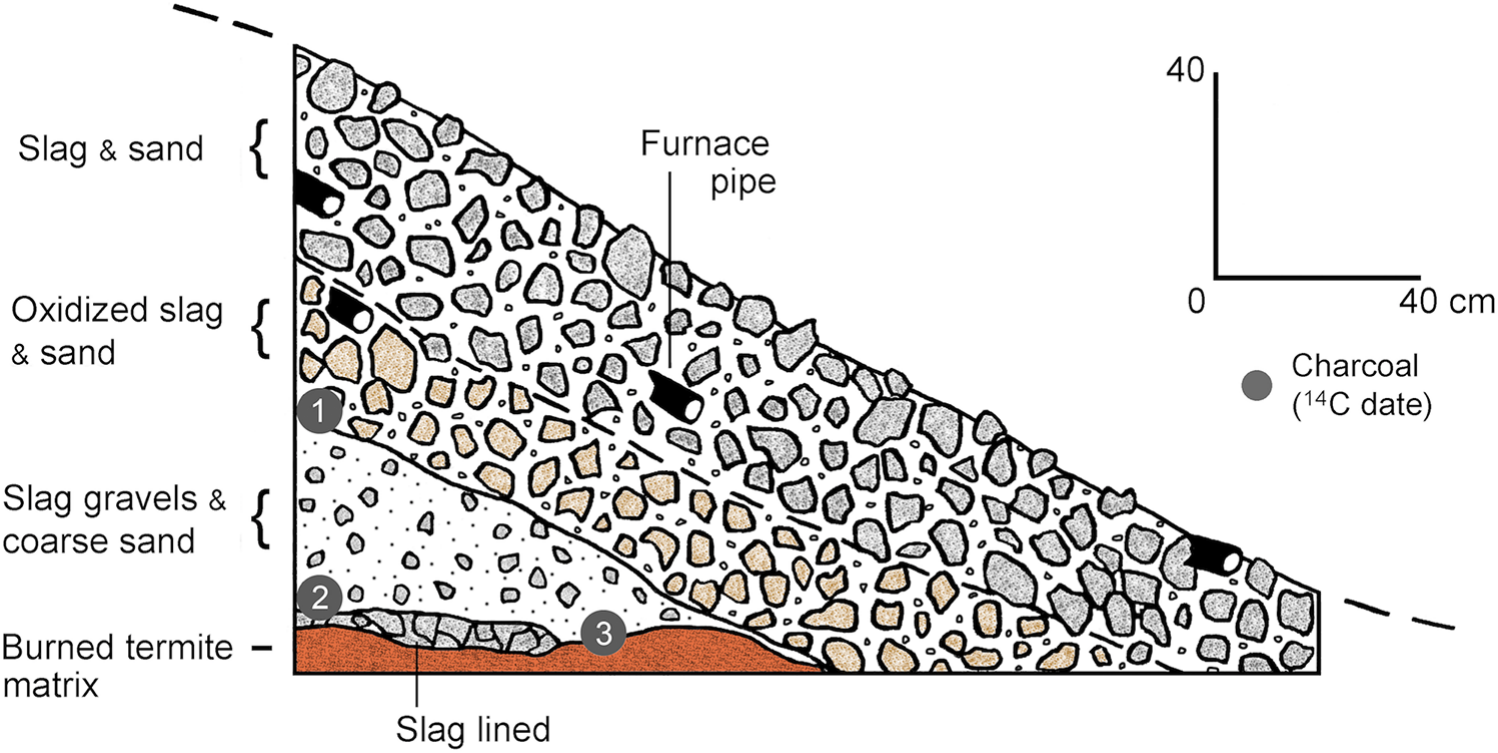
East wall excavation profile in the BB01 slag mound. This profile illustrates the structure typical of most excavated mounds. Radiocarbon dates (cal mid): 1) AD 1795; 2) AD 1795; 3) AD 1804 (see Table 2).

Nineteen mounds of slag were recorded during survey and seven of these features were subject to test excavations (Table 1, Fig 4). Most occur in secondary forest contexts in close proximity to the modern village of Bagbaya and a few others were identified in nearby manioc fields (BB sites; Fig 1, Table 1). The features typically consist of circular or ovate cones of slag with truncated apices that extend 1.5–2 m above the modern ground surface. Eight of the mounds lacked large intrusive trees and/or dense understory vegetation and were sufficiently intact to measure and calculate approximate slag volumes and weights (Table 3). Surface areas measure between 72 and 255 m^2^ with mound (truncated cone) volumes ranging between 39 and 208 m^3^. Slag weight estimates based on mound volumes range from 43,680 kg to over 230,000 kg with a mean of 118,720 kg. Fragments of ceramic furnace pipe (or tuyère) were observed on the surface of most mounds and were appreciably common along mound margins (S1 Fig). All of the tested mounds consist of blocks of slag, slag gravels and sand, including oxidized lenses (Fig 4, S2 Fig). Most contain networks of intrusive roots from trees growing in and around the mounds, and the detritus consists largely of blocky, vesicular slag that appears to have been produced in primary smelting (cf. [72]). In addition to fragmentary tuyère, a number of the excavated cones contained furnace-related remains including pieces of baked clay, burned termite mound fragments, and/or palm nuts, and excavations in five of the mounds also recovered ceramic pot sherds. According to local informants, termite mound matrix and palm nuts were placed in furnaces as part of rituals associated with iron smelting. Termite mounds were also said to have served as furnace foundations and some heated pieces may also represent furnace remnants. Although the mounds were reported by locals to be locations of furnaces and most contained furnace-related remains, no unequivocal structural remnants were identified in test excavations.

**Table 3:** Measurements and approximate slag volumes and weights from a sample of slag mounds in the Bagbaya vicinity.

Site	Length (m)	Width (m)	Height (m)	Surface area (m^2^)	Volume (m^3^)^a^	Weight (kg)^b^
BB01	18	14	1.50	189	98	109,760
BB04	17	16	2.00	204	163	182,560
BB05	17	20	1.75	255	154	172,480
BB07	12	8	1.50	72	39	42,680
BB09	14	14	1.25	147	62	69,440
BB10	20	16	2.50	240	208	232,960
NG01	12	12	1.75	108	63	70,560
OB02	14	12	1.50	126	62	69,440
Means	16	14	1.72	168	106	118,720

^a^Truncated cone volume (π × h × [R^2^ + r^2^ + R × r]/3) multiplied by .80 as sand, roots, etcetera in the tested mounds make up approximately 20% of the matrix.

^b^Based on an air-cooled iron slag weight of 1120 kg/m^3^ [cf. 56].

A number of the slag mounds identified to us by the inhabitants of Bagbaya are named after the families/clans that still occupy the village (e.g., “Aki” [BB10] and “Bokimba” [BB11]) and who claim ownership by virtue of ancestral ties. Most of the locally known and named slag features are clustered within ~one kilometer of the village on the western side of the Lobaye

River (Fig 1). Radiocarbon analyses on charcoal samples from two mounds (BB01 and BB03) returned four date approximations centered on the beginning of the 19^th^ century (cal mid AD 1795–1808; Table 2, Fig 4). Radiocarbon dates of 231±34 (cal mid AD 1719) and 187±32 yr BP (cal mid AD 1799) from neighboring mound BB05 may reflect two different use-episodes, but these dates overlap in the mid-to-late 18^th^ century and more likely represent a period of sustained use.

Additional slag features include an isolated mound on the eastern side of the Lobaye River (ND02), two mounds near the village of NGoula (NG01 and NG02), and two mounds in secondary forest (OB01 and OB02) along the western margin of a wet savanna that contains the OB site complex (Fig 1, Table 1). Although the occupants of Bagbaya were aware of the OB wet savanna and identified this location as their ancestral village, they professed having no knowledge of the associated slag mounds. Site ND02 is a mound named by the Bagbaya villagers and was identified as the oldest known smelting feature purportedly belonging to Ndanga. Radiocarbon assay of charcoal from ND02 excavations yielded a date of 242±34 yr BP (cal mid AD 1664) and this, in fact, represents the oldest age estimate recovered from our sample of dated slag mounds. Charcoal was also collected from excavations in the NG01 and OB slag mounds and each provided more recent age estimates that overlap with dates from the BB mounds; NG01 was dated (cal mid) to AD 1788, and sites OB01 and OB02 date to approximately AD 1808 and AD 1796, respectively.

### Wet Savanna Mounds

Artifact clusters were recorded in two wet savannas (NZ01-5 and OB03-12; Table 1, Fig 1) on the east-side of the Lobaye River within a few kilometers of the mines. Both contained diffuse scatters of pot sherds and slag on and around low sediment mounds that appear to be natural features, including ancient, eroded termite mounds capped by veneers of sand.

The OB complex occurs in an isolated wet savanna identified by the Bagbaya residents as the location of their ancestral village, Ekuba. Excavation units were placed on the gentle slopes of the OB05 and OB06 mounds where surface slag and/or ceramics were observed. Test excavations at OB05 uncovered small clusters of slag, tuyère fragments, and abundant ceramic pot sherds in a charcoal-flecked horizon approximately 25 cm below the ground surface. Radiocarbon assay of two charcoal samples associated with these artifacts returned dates of 131±34 yr BP (cal mid AD 1806) and 188±39 yr BP (cal mid AD 1798) (Table 2). Excavations in OB06 also recovered a few pieces of slag, tuyère, and a single ceramic pot fragment from a buried charcoal-stained layer. A charcoal sample collected from the bottom of the lens dates (cal mid) to approximately AD 1806 and is contemporaneous with the age estimates from OB05. Smooth chunks of baked, brick-like red-brown clay were also recovered at OB06 and appear to represent furnace fragments (cf. [56, 73]). The structure and composition of slag across the OB savanna consists of fluid-like (“coulée”) cast or ladle slag, unlike the blocky slag that predominates at the BB sites. Given the presence of refined cast slag and the overall low abundance of smelting debris in surface and subsurface contexts, it appears that the site witnessed secondary metallurgical processing that may have included the production of “clean” blooms (cf. [74]).

The NZ complex was found in a separate wet savanna identified as “Nzumba” approximately one kilometer east of the Ndanga Grotte (Fig 1). The savanna was known to the Bagbaya villagers because a traditional and well-established foot path from the southern Ndanga mines to the village of Bolembé bisects the savanna. However, the inhabitants of Bagbaya were unaware of any archaeological sites or iron-processing detritus. The NZ complex consists of five separate low mound features containing slag and scattered ceramic sherds. While excavations at NZ06 recovered no subsurface cultural materials, three test units at NZ05 uncovered buried pieces of slag, pottery, tuyère, and/or fire-altered clay deposited in at least two occupations sometime during the 14^th^-to-middle 15^th^ centuries. Radiocarbon analysis of associated charcoal in two units returned dates of 593±34 yr BP (cal mid AD 1354) and 494±34 yr BP (cal mid AD 1424). Excavations in the third test unit also recovered a fractured quartz cobble and pieces of quartz spall that appear to represent a broken hammerstone. Associated charcoal returned a date of 706±35 yr BP (cal mid AD 1320) and provides the earliest age estimate extracted from the 17 Bagbaya radiocarbon samples (Table 2). Similar to the OB features, and regardless of context, the NZ smelting debris is sparsely distributed and includes chunks of dense cast slag that reflect the secondary production of refined iron.

### Sediment Core Analysis

**Fig 5:**
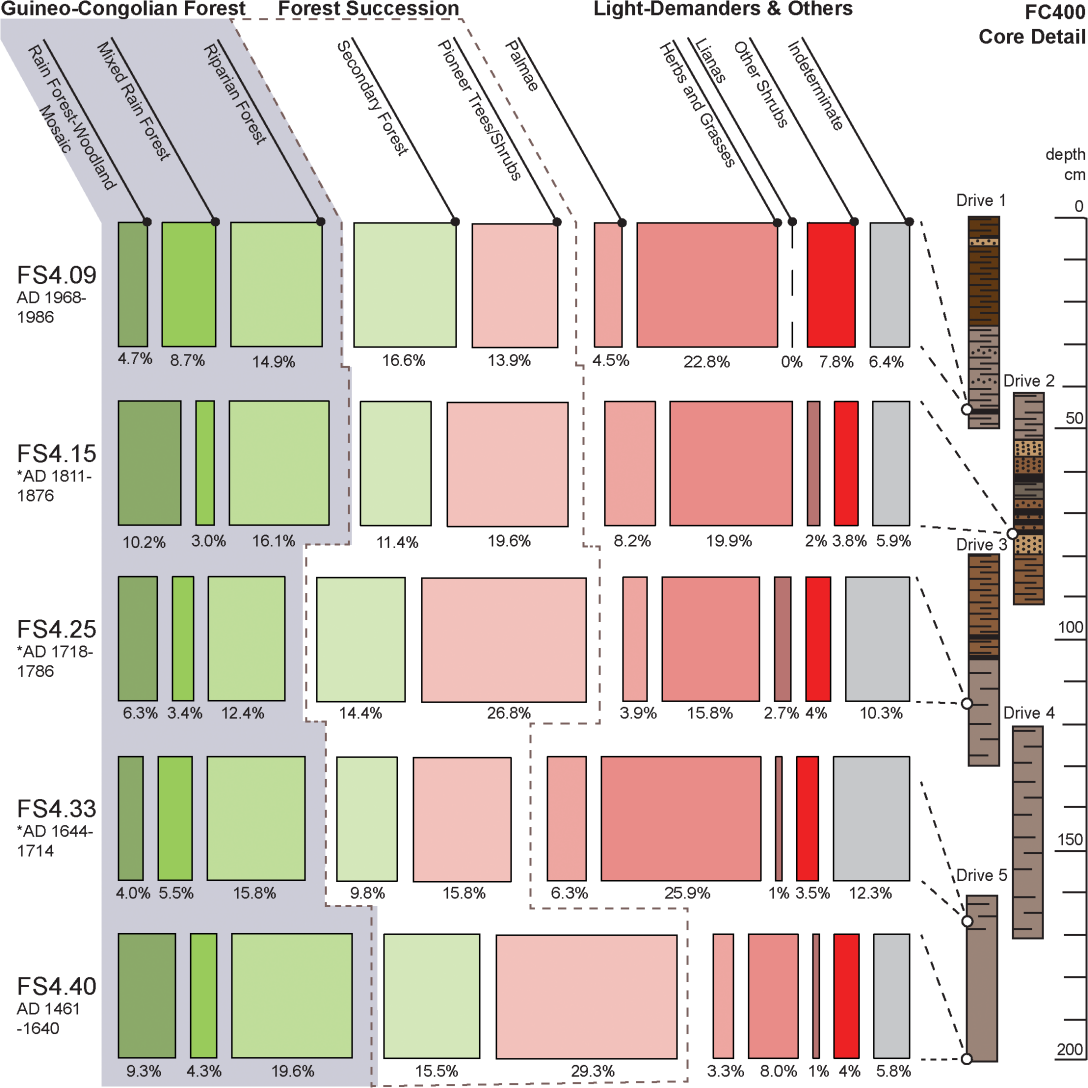
The relative percent of pollen summations in the FC400 sediment samples represented by vegetation types across three broad groups.

Previously published paleoenvironmental data from the NFR region [61, 75] and Mbari Valley [76] demonstrate the utility of riparian sites for reconstructing past environments and our investigations centered on a riparian marsh less than one kilometer from the ND01 and ND03 quarries (Fig 1). Using a portable corer, we retrieved a 2-meter long sedimentary sequence (labeled as FC400) in five overlapping 50 cm-long drives from a filled river channel near the confluence of the Loamé and Lobaye rivers. This site was chosen for sampling because the channel would have captured alluvial deposits, retained sufficient moisture to preserve pollen, and has been protected from scouring during seasonal floods. Here, we report preliminary findings from five organic-rich samples that span the depth and age range of the core (Fig 5). Radiocarbon (AMS) dating and assay of ^210^Pb/^137^Cs indicate that the channel filled in starting after AD 1461–1640 (FS4.40; cal mid AD 1550) and that the upper 50 cm of the core accumulated after AD 1950 (FS4.09).

**Fig 6:**
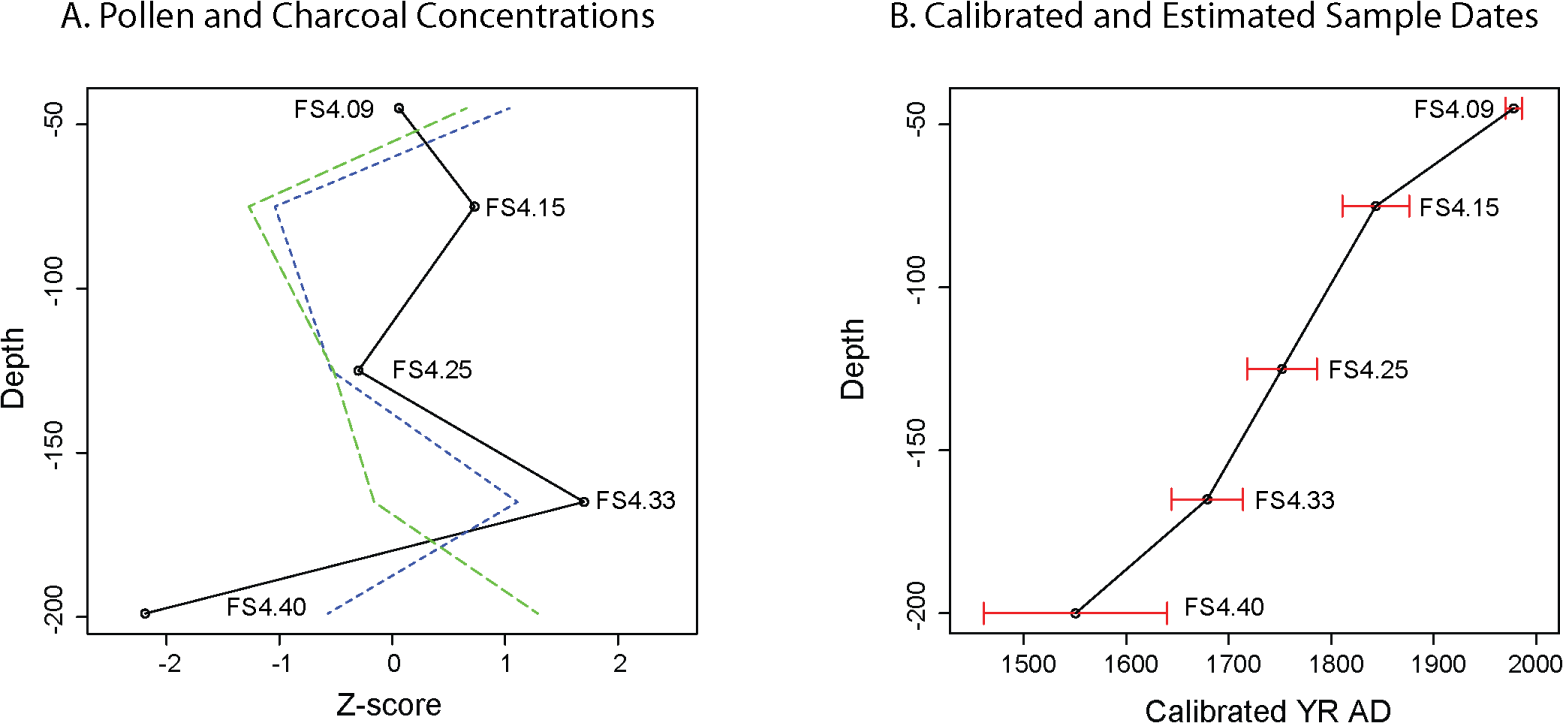
Ages and pollen and charcoal concentrations for the five core samples. A: Plot of pollen, charcoal, and charcoal-pollen ratios as Z-scores. B: Plot of absolute dates and estimated age ranges. Estimates were derived from absolute dates and closely match shifts in pollen concentrations.

Samples from the Loamé core contain well-preserved pollen encased in alluvial/fluvial fine silty sand and mud with each yielding high concentrations of pollen grains (>60e^+10^/g dry wt) of which indeterminate or damaged grains make up less than 15% of the pollen sum (S2 Table). Greater concentration values in the uppermost and lowermost sections of the core (samples FS4.09 and FS4.40) suggest that sedimentation rates were lower in these samples compared to the middle portion of the core (samples FS.4.33, FS4.25, FS4.15: Fig 6A). Based on calibrated absolute dates from assay of ^210^Pb/^137^Cs and ^14^C, 1-cm samples in the upper (< 60 cm) and lower (> 165 cm) reaches of the core represent approximately 2.2 years of deposition. Using a tentative estimate of 1.55–1.8 cm/yr, estimated age ranges were derived for the undated samples (Fig 6B). Clearly, additional analyses will refine this chronology, but these estimates provide a basis to discuss an approximate chronology of vegetation change in the Loamé River coring location and vicinity.

Guineo-Congolian rain forest types and taxa from pioneer and secondary forests make up the majority of the pollen sum in the lowest sampled sedimentary unit (FS4.40), but the more organic-rich deposits above it show a broad decline in forest and increases in herbs and grasses which reach a maximum influx of 25.9% (FS4.33; Fig 5). Sediments from the upper reaches of this depositional unit (FS4.25; circa AD 1750) show a continued declined in Guineo-Congolian forest taxa as pioneer and secondary forest taxa rebound to 14.4% and 26.8%, respectively. Palms experience a modest increase in FS4.33, but decline alongside herbs and grasses in FS4.25 (Fig 5). Together, these samples suggest that between about AD 1460 and 1640 vegetation cover in the Bagbaya area was composed of mixed Guineo-Congolian rain forest showing multiple stages of forest succession and, possibly, colonization of savanna cover. By AD 1714, there was a rather sudden shift to more open forest formations and savanna-woodland cover.

Samples from the more complex sedimentary sequence in the upper 100cm of the core (FS4.15 and 4.09) initially show that Guineo-Congolian forest types experience a moderate rebound in the representation of taxa from riparian and forest-woodland mosaics. Pioneer forest taxa steadily decline in both samples, while mixed rain forests and secondary forest reach a maximum of 8.7% and 16.6% of the pollen sum in FS4.09, respectively. Grasses and herbs are also well-represented in these samples, reaching 22.8% of the pollen sum in sample FS4.09. Bands of dark sediments rich in organic matter and charcoal are common in the middle section of the core, followed by somewhat stable deposition of increasingly organic-rich deposits in the upper 50 cm of the core. These results indicate that after AD 1800 forest-woodland mosaics and riparian forests persist alongside open savanna-woodland vegetation until after AD 1970, when stable secondary and mixed rain forest communities reach their maximum representation in the sampled sediments.

Charcoal concentrations vary widely between FS4.40 and FS4.33 (Fig 6B), moving from a low influx to a high influx between these samples, after which there is a steady decline in charcoal influx until it spikes along with pollen concentrations in FS4.09. The charcoal to pollen ratio corrects for some changes in the depositional environment that would appear to show a spike in charcoal, such as the increase in FS4.09. This ratio indicates that charcoal fluctuations in the lower group of samples may reflect fire-related disturbances followed by an overall reduction in charcoal influx and a muting of sample variability in the upper three samples.

This interpretation is consistent with a previous paleoenvironmental study of a core from the northern edge of the NFR [61], which also identified a shift to open forest formations and a general reduction in microscopic charcoal influx after ~AD 1500. However, that study shows a maximum influx of Poaceae pollen of less than 10% of the pollen sum, while the samples from FS400 show a much more substantial representation of grass pollen. Ostensibly, the reduction in charcoal concentrations in these cores seems at odds with the intensification of prehistoric iron production, but we argue that this pattern reflects a shift in land use practices and the movement of biomass into the sedimentary record. Because iron smelting activities produces a sink for forest biomass by completely volatilizing and removing it from a catchment, there should be less biomass available for both natural and anthropogenic landscape fires. Furthermore, the clear increase in the influx of organic material and the number of indeterminate pollen grains in the sampled sediments strongly argues for a rather sudden filling of this once exposed river channel, triggered by the erosion of exposed soil and sediments. The increase in open forest and savanna-woodland formations that take place in the NFR samples during an interval of time when other portions of the Central African forest witness a regeneration and rebound of forest vegetation [2, 77–79]. This rebound is largely associated with a return to more humid and moister conditions after a prolonged dry interval that punctuated the mid-to-late Holocene [1, 2, 4, 7, 8, 80]. Demographic factors such as localized abandonment in response to tribal warfare and to escape slave-raiding likely also played a role in some areas [76]. However, the marked shift in vegetation in the NFR during this interval of time lends additional support to our hypothesis that iron smelting is a contributing factor, causing both a shift in the movement of biomass in the forest system and the opening of previously forested environments during the last half of the second millennium AD.

## Discussion

Archaeological sites near the village of Bagbaya in the southern Central African Republic provide the first evidence of large-scale iron mining and production in this part of the Congo Basin. Although village occupants reported the existence of some 50–60 slag mounds in the area, we were only able to identify 19 but readily acknowledge that the number of sites reported here is probably far less than the actual number in the area. Due to the density of the vegetation and time constraints of this project, our judgmental investigations undoubtedly missed a number of archaeological sites, especially partially eroded slag mounds. Furthermore, contemporary forest occupants often collect slag and tuyère for other uses. Because stone is very limited in the forest, village occupants readily collect and use these materials as sinkers for fishnets, anchors for boats, pot supports in fire hearths, and in the construction of village structures. Additional contributing factors to the reduction in and dispersal of slag include agricultural activities and forest growth.

The data from the NZ site clusters show that iron-mining and production predates the oral history of the Bagbaya residents by several centuries. Given that the ancestors of the Bagbaya residents migrated into the NFR area in the 18^th^ century, it is possible that the earlier dates from the NZ savanna represent iron production by a separate population on a smaller-scale with the probable production of clean blooms. Although the earliest slag mound (ND02) is considerably older than most of the others, dates from the OB savanna site and BB slag mounds all cluster within a 90 to 100 year interval suggesting that after the arrival of the ancestral Bagbayians iron mining and smelting intensified. Despite the remoteness of this location, the area offered exposed seams of iron ore, clay sources for making tuyères, access to fuel, and a transportation line. Although iron ore is an abundant mineral found throughout most of the African continent, deposits are difficult to find in rainforests where dense vegetation and deep sand deposits cover the terrain. The exposed iron ore seam that comprises the ND mines offered one of the very few locations where ore was readily accessible. Although we did not identify a local clay source, village residents readily acknowledged the existence of sources and we observed ceramics being manufactured from local clay as recently as AD 2000. The forests that envelope this area readily provided fuel for smelting and the Lobaye River likely acted as a natural conduit for transporting iron to other locations. However, the area lacks natural stone that could be used as anvils in the forging process. In fact, we found no evidence supporting the existence of foundries, smithing, and/or forging activities at any of the sites and our test excavations did not recover discarded or broken fragments of formed iron objects. We conclude that while iron was smelted at Bagbaya, most of it was smithed elsewhere.

### Precolonial Iron Production in the Lobaye Region

The sites reported here are especially important because they span an interval of time in the Central African rainforest that is largely unknown from historical documents and archaeological remains. Although the Portuguese under Diogo Cão sailed up the Congo River in AD 1483, forested regions of interior central Africa were among the last to be penetrated by foreign explorers, naturalists, and merchants. Based on historical documents, Vansina [81] hypothesized that the influence of the Atlantic slave trade may have reached the lower portions of the Ubangui River by AD 1787 and oral histories from the Lobaye River Basin record that people fled the area in response to slave raiding as early as AD 1800 [65]. As a tributary of the Ubangui, the Lobaye River was first noted in AD 1885 by Captain Gelfin and the lower portion of the river was surveyed the following year [82]. In AD 1892 the first Europeans made commercial trips up the Lobaye to the town of NGotto to trade for ivory, but the river was not completely explored until AD 1901 [66] after the establishment of Bangui as a colonial outpost (circa AD 1889). Even though European and foreign trade items were available in the Congo Basin and some adjacent regions, interior areas such as the Lobaye apparently had little access to these items [64, 65]. Common markers of historic European influence and trade such as manioc (*Manihot esculenta*) and maize (*Zea maize*) did not reach this area until the late 19^th^ century and possibly not before AD 1900 [63, 64]. Clearly, this portion of Central Africa did not remain isolated from the extended demographic and political consequences of different historical events, but the lack of penetration of trade iron and metals in this area sustained pre-colonial economic iron production and indigenous African distribution networks, even as imports were spreading from the coast to the interior [64, 83].

Although historical data are limited, further insight into the pre-colonial period can be derived from Bahuchet's [66] historical ethnography of the Lobaye region derived from early colonial documents and oral histories from the village of Bagandou located approximately 85 km east of Bagbaya (cf. [55]). According to Bahuchet [66], raw iron was one of the principal trade items transported along the Lobaye River for regional consumption during the pre-colonial period. Raw iron was also possibly transported through intermediaries down the Ubangui River to points south and east. During the late 19^th^ century and into the 20^th^ century these same corridors were used to transport palm oil and other products in a regional trade system (cf. [66]).

Blacksmiths were only associated with certain ethnic groups such as Bofi, Gbaya, and the Monzombo. In fact, ethnic Bofi still occupy villages in the northern portion of the NFR. Bofi furnaces were constructed from hand excavated subterranean pits (or a pit-hearth type described by Pole [84]; cf. [85]). Charcoal and ore were placed in the pit, which was covered with earth and wood. Bellows were attached by tuyères and pumped air into the closed system to create sustained heat. Known foundries and/or areas where concentrations of blacksmiths formed so-called artisanal centers were located in the savanna/forest transitional zones north of Bagbaya, and at the confluence of the Lobaye and Obangui rivers south of Bagbaya. But itinerant blacksmiths also traveled to known ore sources to work in villages and acquire raw material. Smelted iron balls or ingots were traded from central locations or markets, such as Bagbaya.

As with many other portions of Africa, finished and raw iron implements served as currency for commercial exchange and as a means of establishing social and matrimonial relations (via bride price) [66]. European iron bars eventually entered local markets and replaced many items of indigenous manufacture, but indigenous iron objects were often preferred over imported items [26] and raw smelted iron continued to be circulated as a form of currency in the Lobaye area well into the 20^th^ century [66].

### Intensity of Iron Production and the Environmental Consequences of Metallurgy

There are several examples in Africa, such as Meroe [86] and the Mema area [29], where large-scale iron mining and smelting resulted in serious environmental repercussions, including the localized reduction of trees. But the impacts resulting from prehistoric metallurgy on vegetation in the Central African rainforest is generally viewed as being negligible even after the production and use of iron became widespread (after 2,200–2,100 years ago [50]). This view is bolstered by conventional models of forest degradation that were based on inaccurate estimates of charcoal production. Traditional archeological approaches evaluating the vegetational impacts of metallurgy in Africa were often built from estimates of fuel requirements given the amount of slag found in archaeological sites. Using data derived from historical documents, oral histories, and volumetric measurements of slag from pre-colonial Dapaa in Ghana, Goucher [26] hypothesized that deforestation in West Africa resulting from iron production in concert with climate change, gave rise to innovative metal production techniques but eventually resulted in an overall decline in production. Her estimates of the number of tree’s required for fuel (based on slag volumes) were subsequently challenged by Fairhead and Leach [25] who not only questioned the then prevailing views of the historical extent of the African rainforest, but also provided new estimates of the numbers of trees required for charcoal based on volumetric slag measurements from the Basseri region in Togo as reported by de Barros [56, 87]. Fairhead and Leach [25] provide estimates of the number of trees (measured in ha) required to produce charcoal for fuel based on different assumptions about tree densities and regeneration rates. These estimates are substantially smaller than those calculated by Goucher’s [26] methods and they conclude that the number of trees as measured by ha needed for fuel would be less than the amount of land cleared annually for slash and burn agriculture (approximately 8 ha per 1 ton of iron). As they readily acknowledge, these estimates are inherently inaccurate and can vary depending on forest composition, tree densities, regeneration rates, ore quality, furnace technology, and the kinds of production activities that characterize the area. But probably the most imprecise assumption that underlies their analyses is that charcoal could be manufactured from any type of wood. Ethnographic and historical records report that charcoal for iron production was traditionally made out of denser types of wood that could burn for longer periods of time [29, 86, 88]. Fairhead and Leach [25] argue that the selective use of hardwood species for charcoal would not necessarily produce widespread deforestation. However, studies among contemporary rural populations in Africa show that charcoal production does impact forest communities, and that human use history even on a small-scale can significantly influence forest structure [89].

Another line of evidence often cited when evaluating the influence of prehistoric metallurgy (and agricultural practices) on rainforest vegetation is paleoenvironmental records derived from aquatic and marine sediment extracted from large catchments. Although many of these records reveal fine-grained changes in vegetational history, linking these changes to specific anthropogenic processes is difficult. Recently, for instance, Bayon and colleagues [8] argued that intensive land-clearance from cultivation, along with demands for fuel for metallurgy, led to widespread deforestation approximately 2500 years ago based on chemical weathering signals (Al/K) in sediment samples from a marine core from the mouth of the Congo River. But records derived from regional sources tend to homogenize and conflate different prehistoric processes across time and space. These records often cannot be directly correlated with known prehistoric processes because of the scale of analysis and a general lack of comparable archaeological data, especially in forested areas of Africa [90].

We argue for the importance of fine-grained and regional scale studies in assessing the influence of different anthropogenic processes across time and space. The scale of iron production reported here is comparable to other known iron production areas in Africa. Again, using the mean volume and weight estimates for the eight intact features we measured (Table 3), the 19 identified mounds contain some 2015 m^3^ of slag weighing in excess of 2,250,000 kg. If we assume that 50 mounds exist, as reported by the inhabitants of Bagbaya, then it is possible that 5,300 m^3^ of slag was produced during the ~100 years that the area witnessed intensive production. Comparative data in Table 4 shows that these values (as measured on a 100-year scale) are smaller than some, but they do overlap with other sites where intensive iron production occurred, including Dapaa in Ghana [26] and Bassar (Period 2) in Togo [56]. The consequences of this level of production for vegetation cover in the Bagbaya region is visible in the microbotanical evidence, which shows a broad reduction in forest taxa in favor of grasses and herbs during the 18^th^and 19^th^ centuries AD. This signals a broadly distributed pattern of forest disturbance across forest types and successional stages that encouraged spatial heterogeneity in vegetation cover and the development of wet savanna ‘islands’ in otherwise forested areas.

**Table 4:** Comparisons of reported slag volumes in late Holocene sites in West Africa.

Site	Total volume (m^3^)	Span of years	100 yr. rate (m^3^)
Bassar (Period 2)^a^	13,260	AD 1300–1600 (300 yr.)	4420
Bassar (Period 2)^a^	40,893	AD 1550–1800 (350 yr.)	11,684
Bassar (period 3)^a^	28,172	AD 1800–1925 (125 yr.)	22,538
Dapaa^b^	1962	AD 1400–1700 (300 yr.)	654
Ndop Plain^c^	163,000–133,000	AD ~1800’s (100 yr.)	163,000–133,000
Bagbaya^d^	2015–5,300	AD 1719–1808 (~100 yr.)	2015–5300

^a^Northern Togo [56].

^b^Ghana [26].

^c^Cameroon [91, 92].

^d^Central African Republic (this study). The smaller value of 2015 m^3^ reflects a volumetric estimate for the 19 mounds reported here. The larger value of 5,300 m^3^ is an estimate of slag for 50 mounds assuming the average mound size is 106 m^3^.

It is difficult to place the Bagbaya sites in a regional context because in forested regions of central Africa, such as the NFR, very little comparable archaeological research has been conducted. The data reported here constitute some of the first systematic archaeological and paleoenvironmental research ever conducted in this portion of the Congo Basin. We contend that iron production areas, such as those represented by the features at Bagbaya, would have utilized forest biomass for charcoal production which likely had significant and unique impacts on local vegetation communities compared to swidden agriculture. Local-scale impacts such as those associated with iron production at Bagbaya may have been very common in rainforest habitats where iron ore is often only exposed as localized outcrops and/or scattered boulders and can be difficult to find. We suggest that the impacts of iron production on rainforest vegetation communities cannot be excluded based solely on microfossil evidence and can only be adequately evaluated when plant remains are examined in concert with local archaeological and historical evidence.

## Supporting Information

S1 Fig Example of in-situ furnace pipe (tuyère) fragments on the margin of a slag mound (Site BB05). The wooden trowel handle below the tuyère measures 9.2 cm in length; photo by DN Schmitt.
(TIF)

S2 Fig View north of test excavations in the NG01 slag mound at approximately 70 cm below surface. Radiocarbon assay of charcoal from 60 cm below surface returned a date of 217±48 yr BP (Table 2). Note the oxidized (orange) slag and sand in the lower portions of the unit; photo by KD Lupo.
(TIF)

S1 Table Specimen numbers, provenience, and repository information for collected artifacts and sediment samples. (PDF)

S2 Table Summary of major pollen groups and selected taxa as a percent of the total pollen sum in each sample. (PDF)

S1 Text Résumé (French abstract). (PDF)
