# Supplementary figures and images for: On Intensive Late Holocene Iron Mining and Production in the Northern Congo Basin and the Environmental Consequences Associated with Metallurgy in Central Africa
Source: PLoS One. 2015 Jul 10;10(7):e0132632. doi: 10.1371/journal.pone.0132632 (PMC4498739; doi:10.1371/journal.pone.0132632)

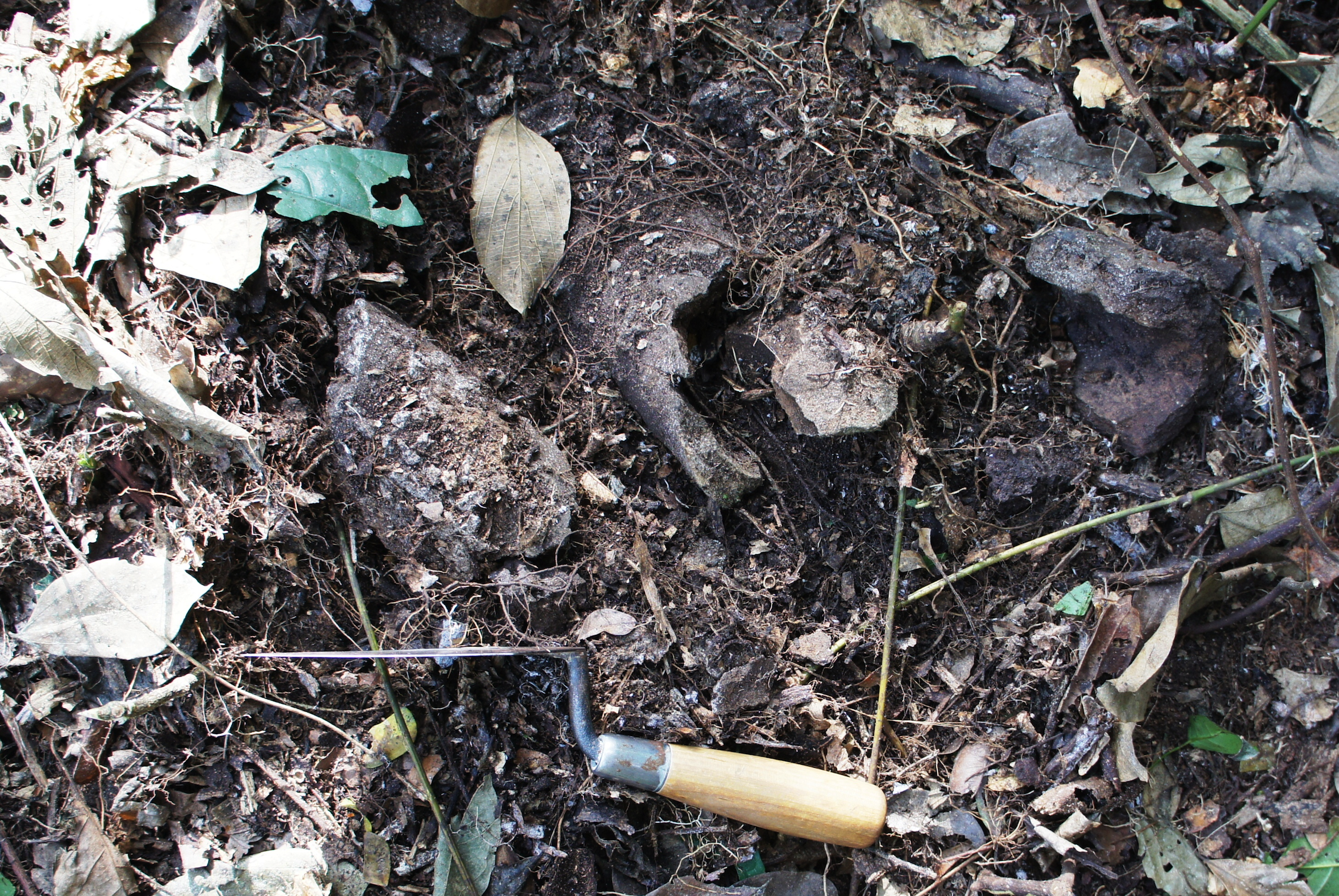

Supplement: S1 Fig — The wooden trowel handle below the tuyère measures 9.2 cm in length; photo by DN Schmitt. (TIF) [file pone.0132632.s001.tif]

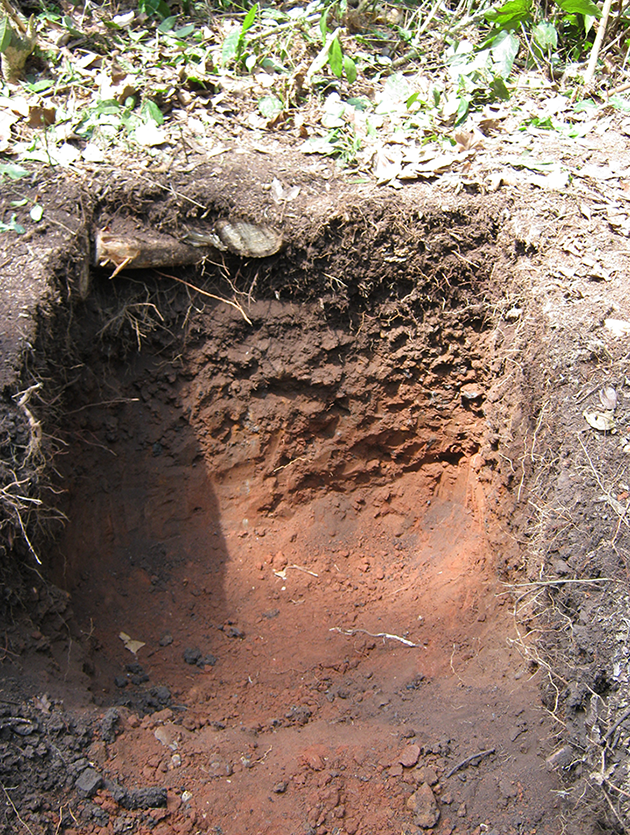

Supplement: S2 Fig — Radiocarbon assay of charcoal from 60 cm below surface returned a date of 217±48 yr BP (Table 2). Note the oxidized (orange) slag and sand in the lower portions of the unit; photo by KD Lupo. (TIF) [file pone.0132632.s002.tif]
