# Supplementary material for: On Intensive Late Holocene Iron Mining and Production in the Northern Congo Basin and the Environmental Consequences Associated with Metallurgy in Central Africa
Source: PLoS One. 2015 Jul 10;10(7):e0132632. doi: 10.1371/journal.pone.0132632 (PMC4498739; doi:10.1371/journal.pone.0132632)
Supplement: S1 Table — (PDF) [file pone.0132632.s003.pdf]

**S1 Table. Specimen numbers, provenience, and repository information for collected artifacts and sediment samples.**

| Site | Field specimen no. | Description | Unit no. (depth below surface [cm]) | Status[a] |
|---|---|---|---|---|
| ND01-E | 1 | Quartzite | 1 (0-30) | 3 |
| ND01-E | 2 | Tuyère fragment | 1 (53) | 1 |
| ND01-E | 3 | Charcoal | 1 (55) | 2 |
| ND01-G | 4 | Charcoal | 2 (21) | 1 |
| ND01-G | 5 | Quartzite and ceramic fragments | 2 (0-50) | 1 |
| NZ01 | 6 | Tuyère fragments | (Surface) | 3 |
| NZ01 | 7 | Ceramic fragment | (Surface) | 1 |
| NZ03 | 8 | Tuyère and ceramic fragments | (Surface) | 1 |
| NZ04 | 9 | Tuyère fragments | (Surface) | 3 |
| NZ02 | 10 | Ceramic fragment | (Surface) | 1 |
| NZ03 | 11 | Charcoal | 1 (12) | 1 |
| NZ03 | 12 | Charocal | 1 (33) | 2 |
| NZ03 | 13 | Slag sample | 1 (0-33) | 3 |
| NZ03 | 14 | Tuyère and ceramic fragments | 1 (0-30) | 1 |
| NZ03 | 15 | Tuyère fragments | 1 (0-33) | 1 |
| NZ03 | 16 | Tuyère and ceramic fragments | 3 (0-50) | 1 |
| NZ03 | 17 | Charcoal | 3 (27) | 2 |
| NZ03 | 18 | Charcoal | 3 (15) | 1 |
| NZ03 | 19 | Tuyère fragments | 3 (15) | 1 |
| NZ03 | 20 | Tuyère fragments | 4 (Surface) | 1 |
| NZ03 | 21 | Tuyère fragments | 2 (0-28) | 1 |
| NZ03 | 22 | Tuyère fragments | 4 (19) | 3 |
| NZ03 | 23 | Charcoal | 4 (29) | 2 |
| BB01 | 26 | Tuyère fragments and slag sample | 1 (0-90) | 1 |
| BB01 | 27 | Slag sample | 1 (130) | 3 |
| BB01 | 28 | Palm nuts | 1 (90) | 1 |
| BB01 | 29 | Charcoal | 1 (50) | 2 |
| BB01 | 30 | Charcoal | 1 (90) | 2 |
| BB01 | 31 | Charcoal | 1 (112) | 2 |
| BB05 | 32 | Charcoal | 1 (24) | 2 |
| BB05 | 33 | Ceramic fragments | 1 (0-24) | 1 |
| BB01 | 34 | Tuyère fragments and assoc. sediment sample | 1 (0-90) | 3 |
| BB03 | 35 | Tuyère fragments and assoc. sediment sample | 1 (20) | 3 |
| BB03 | 36 | Charcoal | 1 (24) | 2 |
| BB03 | 37 | Ceramic fragments | 1 (Surface) | 1 |
| BB05 | 38 | Tuyère fragments and assoc. sediment sample | 1 (30-50) | 3 |
| BB05 | 39 | Charcoal | 1 (34) | 1 |
| BB05 | 40 | Ceramic fragments | 1 (40) | 1 |
| BB05 | 41 | Charcoal | 1 (61) | 2 |
| OB | 42-46 | Isolated ceramic fragments | (Surface) | 1 |
| OB02 | 47 | Tuyère fragments and slag sample | 1 (Unknown) | 3 |
| OB02 | 48 | Sediment sample | 1 | 3 |

| | | | | |
|---|---|---|---|---|
| OB02 | 49 | Charcoal | 1 (25) | 2 |
| OB05 | 50 | Ceramic fragments | 2 (14-18) | 1 |
| OB05 | 51 | Charcoal | 2 (18) | 1 |
| OB05 | 52 | Ceramic fragments | 2 (Surface) | 1 |
| OB05 | 53 | Charcoal | 2 (22) | 2 |
| OB05 | 54 | Charcoal | 2 (25) | 2 |
| OB05 | 55 | Ceramic fragments | 2 (16-22) | 1 |
| OB05 | 56 | Ceramic fragments | 1 (Surface) | 1 |
| OB05 | 57 | Ceramic fragments | 2 (Surface) | 1 |
| OB05 | 58 | Ceramic fragments | 2 (20-25) | 1 |
| OB05 | 59 | Charcoal | 2 (28-30) | 1 |
| OB | 60 | Isolated ceramic fragment | (Surface) | 1 |
| OB06 | 61 | Ceramic fragments | 1 (Surface) | 1 |
| OB06 | 62 | Tuyère fragments and assoc. sediment sample | 1 (17) | 3 |
| OB06 | 63 | Charcoal | 1 (28) | 2 |
| OB06 | 64 | Charcoal | 1 (27) | 1 |
| OB01 | 65 | Tuyère fragment and assoc. sediment sample | 1 (18) | 3 |
| OB01 | 66 | Charcoal | 1 (17) | 1 |
| OB01 | 67 | Charcoal | 1 (33) | 2 |
| BB14 | 68 | Sediment sample | (0-10) | 3 |
| BB14 | 69 | Tuyère fragments and slag sample | (0-10) | 1 |
| NG01 | 70 | Ceramic and baked clay fragments | 1 (Surface) | 1 |
| NG01 | 71 | Charcoal | 1 (39) | 1 |
| NG01 | 72 | Charcoal | 1 (43) | 1 |
| NG01 | 73 | Tuyère fragments and assoc. sediment sample | 1 (48) | 3 |
| NG01 | 74 | Charcoal | 1 (60) | 2 |
| NG01 | 75 | Tuyère fragments and assoc. sediment sample | 1 (63) | 3 |
| ND02 | 76 | Charcoal | 1 (74) | 2 |
| ND02 | 77 | Tuyère fragments and assoc. sediment sample | 1 (75) | 3 |
| ND02 | 78 | Ceramic fragments | (Surface) | 1 |
| Core[b] | 4.01-4.40 | Sediment samples | 0-200 cm | 1 |

[a]1 = Housed at the Department of Anthropology, Southern Methodist University, Dallas, Texas; 2 = destroyed in $^{14}C$ assay; 3 = collections housed at the Centre Universitaire de Recherche et de Documentation en Histoire et Archeologie Centraficaines, Université de Bangui, Central African Republic that were stolen or destroyed during the coup d'état of 2013.
[b]Includes sub-samples of the original sediment samples that were partially processed for this and other projects.
