# Supplementary material for: On Intensive Late Holocene Iron Mining and Production in the Northern Congo Basin and the Environmental Consequences Associated with Metallurgy in Central Africa
Source: PLoS One. 2015 Jul 10;10(7):e0132632. doi: 10.1371/journal.pone.0132632 (PMC4498739; doi:10.1371/journal.pone.0132632)
Supplement: S2 Table — (PDF) [file pone.0132632.s004.pdf]

S1 Table. Summary of major pollen groups and selected taxa as a percent of the total pollen sum in each sample.

GROUPS/TAXA	FAMILY	FS4.40	FS4.33	FS4.25	FS4.15	FS4.09
PALMAE - ARECACEAE		**3.26**	**6.28**	**3.89**	**8.19**	**4.46**
Borassus/Hyphaene	PALMAE	0.5	0.25	0.73	3.23	0.99
Elaeisguineensis	PALMAE	0.75	1.76	0.73	1.24	1.49
Phoenix	PALMAE	0	0	0.49	1.49	0
Raphia	PALMAE	2.01	2.76	1.7	1.24	1.73
HERBACEOUS		**8.02**	**25.88**	**15.82**	**19.85**	**22.77**
Poaceaeundiff.	POACEAE	3.51	16.08	8.52	12.66	16.34
Poaceae-Damaged	POACEAE	0.75	4.52	1.46	4.22	1.24
Cyperaceaeundiff.	CYPERACEAE	1.75	2.51	4.38	1.49	2.97
LIANAS		**1.01**	**1.00**	**2.68**	**1.98**	**0.00**
Stephania/Tiliacora	MENISPERMIACEAE	0.5	1.01	2.68	1.49	0
GCRF-WOODLAND MOSAIC		**9.27**	**4.02**	**6.33**	**10.17**	**4.7**
Blighia	SAPINDACEAE	2.01	0.25	0.97	3.23	0.25
Vitex-type	VERBENACEAE	0.5	0.5	1.95	2.23	0.5
MIXED GC RF		**4.26**	**5.53**	**3.41**	**2.98**	**8.66**
Antiaris/Milicia/Morus	MORACEAE	1.25	2.01	0.24	0.25	2.97
Olax	OLACACEAE	0.25	0.5	0.97	0.99	2.72
PIONEER TREE-SHRUB		**29.32**	**15.83**	**26.76**	**19.6**	**13.86**
Lannea/Sclerocarya	ANACARDIACEAE	3.01	0.5	1.95	1.74	0.25
Macaranga/Mallotus/Mareya	EUPHORBIACEAE	5.26	3.52	4.38	2.73	1.73
Alchornea	EUPHORBIACEAE	9.02	4.27	11.92	5.21	8.91
Nauclea-type	RUBIACEAE	4.26	4.27	3.89	3.97	0.5
Trema-type orientalis	ULMACEAE	3.01	0.25	0.97	1.74	0.25
RIPARIAN GC RF		**19.55**	**15.83**	**12.41**	**16.13**	**14.85**
Entada/Prosopis/Piptadeniastrum	FABACEAE - MIMOSACEAE	2.01	0.75	0.49	0.74	2.23
Irvingia/Klainedoxa	IRVINGIACEAE	1.5	0	1.22	2.23	0.5

Lophira	OCHNACEAE	1.25	2.26	1.7	2.48	1.98
Uapaca	PHYLLANTHACEAE	11.78	8.79	2.68	6.7	5.45
SECONDARY FOREST		**15.54**	**9.8**	**14.36**	**11.41**	**16.58**
Terminalia	COMBRETACEAE	3.01	0.75	1.95	0.99	2.97
Celtis	CANNABACEAE	2.51	3.77	4.62	3.47	3.22
Musanga/Myrianthus	MORACEAE	9.02	3.52	6.33	5.96	8.42
VARIOUS		**4.01**	**3.51**	**4.01**	**3.76**	**7.77**
Moraceaeundiff.	MORACEAE	2.01	0.25	0	0.25	4.21
Sapotaceaeundiff.	SAPOTACEAE	0.5	0.5	0.49	0.25	0.5
INDETERMINATE		**5.76**	**12.32**	**10.33**	**5.93**	**6.35**
