# Supplementary material for: On Intensive Late Holocene Iron Mining and Production in the Northern Congo Basin and the Environmental Consequences Associated with Metallurgy in Central Africa
Source: PLoS One. 2015 Jul 10;10(7):e0132632. doi: 10.1371/journal.pone.0132632 (PMC4498739; doi:10.1371/journal.pone.0132632)
Supplement: S1 Text — (PDF) [file pone.0132632.s005.pdf]

Résumé

Une question en coursdans les reconstitutions paléoenvironnementales de la forêt d'Afriquecentrale concerne le rôleque la métallurgie préhistorique a joué dans l'élaboration de la vegetation forestière. Nous rapport onsici la preuve de l'exploitation minière intensive de minerai de feret de la fonte dans les regions forestières du nord du bassin du Congo datant de l'Holocène. Estimations volumétriques sur extrait du minerai de fer et des monticules de scories associés de sites préhistoriques dans le sud de la République centrafricaine suggèrent la production de fer à grandee chelle à égalité avec d'autres domaines de fabrication fer archéologiques et historiquement connus. Ces données documentent la première preuve d'exploitation du feret la production intensive couvrant environ 90 ans avant l'occupation coloniale (circa AD 1889) et pendant un intervalle de temps qui est mal représenté dans l'enregistrement archéologique. Les zones de site supplémentaires pré-datant ces restes par 3-4 siècles reflètent une période antérieure de la production de fer sur une plus petite échelle. Micro-botanique preuves d'un ecarotte de sediments recueillis à partir d'un piège riveraine adjacentein dique une réduction des arbres d'ombrage adapté de concert avec une augmentation des espèces héliophiles couvrant l'intervalle de temps associé à l'intensification de ferminier. Ce changement se produit pendant le même intervalle de temps quand beaucoup portions de l'Afrique central ontassisté transgressions forestières associées à un retour à des conditions plus humides commençant ily a 500 à 100 ans. Bien que les donné es présentée sici ne démontrent pas que les activités de fonderie de fer a causé des changements de la vegetation répandue en Afriquecentrale, nous sou tenons quel'exploitation minière et la fonte intense peuvent avoir les impacts localisé et potential le mentrégion aux sur les communautés végétales. Ces donné es démontrent en outre la valeur haute d'associer les analyses archéologique et paléoenvironnementales de reconstituer l'histoire de la forêt à l'échelle régionale.
